# The Readiness for Return to Work Scale; Does it Help in Evaluation of Return to Work?

**DOI:** 10.1007/s10926-021-10009-4

**Published:** 2021-10-16

**Authors:** Lene Aasdahl, Marius Steiro Fimland, Cecilie Røe

**Affiliations:** 1 Department of Public Health and Nursing, Faculty of Medicine and Health Sciences, Norwegian University of Science and Technology, NTNU, MTFS, Postboks 8905, 7491 Trondheim, Norway; 2 Unicare Helsefort Rehabilitation Centre, Rissa, Norway; 3 Department of Neuromedicine and Movement Science, Norwegian University of Science and Technology, Trondheim, Norway; 4 Department of Physical Medicine and Rehabilitation, St. Olavs Hospital, Trondheim University Hospital, Trondheim, Norway; 5 Department of Physical Medicine and Rehabilitation, Oslo University Hospital, Oslo, Norway; 6 Institute of Clinical Medicine, University of Oslo, Oslo, Norway

**Keywords:** Sick leave, Musculoskeletal diseases, Mental health, Rehabilitation

## Abstract

*Purpose* The Readiness for Return to Work (RRTW) scale is used to evaluate workers’ readiness to resume work after sick leave. Previous research has questioned the RRTW scale’s constructs and stages. The aim of this study was to assess the unidimensionality of the RRTW scale and its six subscales by evaluating its fit to the Rasch model, and furthermore to assess if Rasch-based scaling would improve its predictive value, compared with the conventional use of the scale. *Methods* A prospective cohort study with 12 months of follow-up. Individuals (*n* = 397) sick-listed due to musculoskeletal, unspecified, or common mental health disorders undergoing rehabilitation were included: 191 were full-time sick-listed (not working), and 206 were part-time sick-listed (working). A Rasch analysis was applied to evaluate the measurement properties of the RRTW scale in the working and not working participants at baseline. Linear and logistic regressions were used to assess how well Rasch-based scaling predicted future work participation during the 12 months of follow-up. *Results* The RRTW subscales had too few items to represent underlying dimensions properly, and the items fitted poorly within the subscales. A constructed variable based on the items that fit together for not working individuals poorly predicted future work participation. The individuals’ scores across stages were disordered, indicating a lack of ordered stages. *Conclusions* This study reveals poor measurement properties of the Norwegian version of the RRTW scale in individuals with musculoskeletal and common mental disorders, with neither the subscales nor the stages closely associated with return to work.

**Supplementary Information:**

The online version contains supplementary material available at 10.1007/s10926-021-10009-4.

## Introduction

The Readiness for Return to Work (RRTW) model is one of many models that attempt to describe the complex process of returning to work after a long-term sickness absence [1, 2]. In this model, the return-to-work (RTW) process is described as consisting of several stages that the sick-listed worker progresses through on the way to sustainable RTW [1]. The model was designed to measure not only the agency of the worker but also how the health care system, the workplace, and the insurance system affect the sick-listed worker’s progress towards RTW [1]. It has been suggested that the model might help professionals working with sick-listed workers identify which stage the individual is on and assist the worker accordingly [3, 4].

The RRTW scale was developed by Franche et al. [4] in order to improve interventions targeting RTW. The original version comprised 34 items, of which 22 were applied to define six stages of change (subscales). In a Canadian validation study, Franche et al. [4] identified four stages of change for those not working: (a) Precontemplation: the person is not thinking of behavior change in terms of RTW; (b) Contemplation: the person has started to think about RTW but is ambivalent and is not actively engaged in making concrete plan for RTW; (c) Prepared for action—self-evaluative: the person actively seeks information and makes concrete plans for RTW; and (d) Prepared for action—behavioral: the person sets the RTW plan into action. They identified two stages for those working: (a) Uncertain maintenance: the person has returned to work but experiences challenges in staying at work and (b) Proactive maintenance: the person has developed good strategies for staying at work. Franche et al. [4] described two approaches for scoring the questionnaire: (a) the multidimensional approach, recommended for research, where a mean score is calculated for all the different subscales, and (b) the stage allocation approach, recommended for clinical use, where the individual is allocated to the stage where they have the highest mean score. When subscales had similar mean scores, Franche et al. [4] allocated the individual to the lowest stage.

Later studies have had difficulty confirming the original four-plus-two factor structure proposed by Franche et al. [4]. In a Norwegian study, Braathen et al. [3] identified two factors for those not working and two factors for those working. A Danish study confirmed neither the original Canadian nor the Norwegian scoring model, and the results suggested a possible floor effect for precontemplation [5]. In another Canadian study, Park et al. [6] also did not confirm the original structure; they found only three factors for those not working. In a previous prospective cohort study, we found that high scores on the most advanced dimensions towards RTW were not the ones that predicted work outcomes best [7]. With the multidimensional approach, the association was stronger for higher scores on the Prepared for action—self-evaluative dimension than the Prepared for action—behavioral dimension, which is contrary to the order in the model. With the stage allocation approach, we found that those allocated to the Uncertain maintenance stage had a higher probability of sustainable RTW than those allocated to the Proactive maintenance stage, also contradictory to the model. Furthermore, stage allocation was problematic due to several ties between not necessarily adjacent dimensions in the RTW process [7]. Hence, more research is needed on the RRTW construct.

Despite ordinal scaling of the items, the psychometric properties of the RRTW scale have so far not been evaluated with Rasch analysis. The advantage of the Rasch approach compared with conventional factor analysis is its lack of assumption of equal intervals for the scoring options and its use of parametric based statistics. In addition, the Rasch approach allows for evaluation of whether the scale works in the same way across different populations.

It has been suggested that future studies on the RRTW scale should look at the initial pool of items for the scale and not just those included in the final version of the scale. Hence, the aim of this study was to assess the unidimensionality, evaluated by fit to the Rasch model, of the RRTW scale and its six subscales using Rasch analysis and the initial pool of items. Furthermore, we wanted to assess if Rasch-based scaling would improve the predictive value of the scale regarding sickness absence and RTW over one year.

## Materials and Methods

### Study Design

This study applied a prospective cohort design with 12 months of follow-up. Participants took part in one of three randomized controlled trials evaluating the effects of occupational rehabilitation on sickness absence [8, 9]. Two randomized trials compared different inpatient occupational rehabilitation programs with outpatient acceptance and commitment therapy [8]. The third trial compared inpatient programs with or without an added workplace intervention [9]. Several articles have been published from these projects, so the methods therefore overlap somewhat [10–18]. The studies were approved by the Regional Committee for Medical and Health Research Ethics in Central Norway (No. 2012/1241 and 2014/2279).

### Participants

Common eligibility criteria for the trials were individuals aged 18 to 60 years who had been sick-listed for 2 to 12 months (at least 50% if on part-time sick leave) with a diagnosis within the musculoskeletal (L), psychological (P), or general and unspecified (A) chapters of ICPC-2 (International Classification of Primary Care, Second Edition). In addition, potential participants in the third trial had to be employed at least 20%, have an employer, and anticipate at least four more weeks of sick leave. Common exclusion criteria were: (a) alcohol or drug abuse; (b) serious somatic (e.g., cancer and unstable heart disease) or psychiatric disorders (e.g., high suicide risk, psychosis, and ongoing manic episodes); (c) disorders requiring specialized treatment; (d) pregnancy; (e) current participation in another treatment or rehabilitation program; (f) insufficient oral or written Norwegian language skills to participate in group sessions and fill out questionnaires; (g) scheduled for surgery within the next 6 months; and (h) serious problems with functioning in a group setting.

### The Rehabilitation Programs

The inpatient occupational rehabilitation programs were multicomponent programs designed to target three areas of rehabilitation: mental training, physical training, and work-related problem-solving [8, 9]. The inpatient programs consisted of acceptance and commitment therapy, individual and group-based physical exercise training, mindfulness, psychoeducation on various topics, and individual meetings with a coordinator to solve work-related problems and create an RTW plan. Acceptance and commitment therapy is a form of cognitive behavioral therapy, where both negative and positive experiences are accepted [19]. The individual’s values should guide their actions towards their goals, and the aim is to increase psychological flexibility through mindfulness techniques, values, and committed action [18–20]. In the first two trials the program lasted 3.5 weeks and 4 + 4 days (with 2 weeks at home in between). In the third trial, the inpatient programs lasted 2 + 1 weeks with 1 week at home in between. The content of the programs was similar to the program described above. In addition, during the week at home, one of the groups received a workplace intervention. The workplace intervention consisted of (a) a preparatory part; (b) a workplace meeting involving the sick-listed worker, the employer, and the primary rehabilitation therapist at the rehabilitation center; and (c) follow-up work related to the meeting. In contrast to the inpatient programs, the outpatient program targeted mainly the mental aspect of the RTW process. The program consisted of outpatient acceptance and commitment therapy, and participants were offered 2.5-h group sessions once weekly for 6 weeks. In addition, one group session provided psychoeducation on physical activity, two individual sessions featured a social worker to clarify personal values and work-related issues, and a short individual closing session was attended by the social worker and the group therapist (a psychologist or a medical doctor). In the last meeting a summary letter was written to the participant’s general practitioner. Primary outcomes in the randomized controlled trials were the number of sickness absence days and the time to sustainable RTW. The 3.5-week program reduced sickness absence compared with the outpatient program [12], while there was no evidence for an effect of the 4 + 4 days program [10] or the workplace intervention [11].

### Study Context

All legal residents are included in the Norwegian public insurance system. Medically certified sick leave is compensated with 100% coverage for the first 12 months (with some limitations based on salary). The first 16 days are covered by the employer, while the rest is covered by the Norwegian Labor and Welfare Administration (NAV). After 12 months it is possible to apply for more long-term medical benefits, which cover approximately 66% of one’s income.

### Questionnaires

The RRTW scale [4] consists of two parts: Part A features 22 questions to be answered by participants who are 100% sick-listed, whereas working participants (including those on part-time sick leave) answer Part B (12 questions). In the revised versions of the RRTW scale, fewer number of questions are used to define the stages of Precontemplation (items A1, A4, and A22), Contemplation (A15, A20, and A21), Prepared for action—self-evaluative (A9, A12R [reversed item scale], A13, and A18), and Prepared for action—behavioral (A6, A10, and A11) in the not working sample, and the stages of Uncertain (B8, B9, B10, B11R, and B12) and Proactive maintenance (B2, B5, B6, and B7) in the working sample (Tables 2 and 3). In the Norwegian version applied in the present study, the questions are rephrased with proper nouns (first person) [21]. The wording of two questions are also changed: from *pain* and *injury* in the original scale to *health complaints* in the Norwegian cross-cultural adaption of the scale [21]. All 34 items are scored on a 5-point Likert scale, from 1 *(strongly disagree*) to 5 (*strongly agree*). For 25 of the 34 items, *strongly disagree* indicates that returning or maintaining work is unlikely, while for the remaining nine items it indicates that doing so is likely (i.e., reversed). All participants completed the RRTW scale via internet-based questionnaires before they started the rehabilitation programs (baseline).

### Sick Leave Register Data

Sick leave was measured using data from the Norwegian Labor and Welfare Service, where all individuals receiving any form of sick leave or disability benefits in Norway are registered. The data comprised all registered medical benefits individually traceable for each participant by their social security number. Two work measures were constructed: (a) the number of sickness absence days, defined as the number of days receiving medical benefits during 12 months of follow-up after inclusion (part-time sick leave was recalculated as whole sick leave days), and (b) sustainable RTW, defined as 1 month without receiving medical benefits during follow-up.

### Statistical Analysis

A Rasch analysis [22, 23], was applied to evaluate the measurement properties of the RRTW scale for the working and not working subset at baseline (start of rehabilitation). The Rasch analysis was applied to all 22 items in the not working group, followed by an analysis of the 13 items included in the four subscales, and lastly, a separate analysis of the subscales of Precontemplation (three items), Contemplation (three items), Prepared for action—self-evaluative (four items), and Prepared for action—behavioral (three items). The same procedure was followed for the working group—analyzing all 12 items in the working group, followed by an analysis of the nine items generally constituting the Uncertain (five items) and Proactive maintenance (four items) dimensions, and lastly, a separate analysis of the five items of the Uncertain and the four items of the Proactive maintenance dimensions. All items initially scored on a 5-point scale were analyzed regarding the thresholds between the scoring points/levels. The scoring options were reversed for the negatively framed questions (A1, A4, A12, A16, A22, B8, B9, B10, and B12). Hence, for all items a score of 5 represented the highest likelihood of returning to and maintaining work. If the thresholds were disordered (i.e., the score levels did not separate the level of the underlying construct), the responses were re-scored. Local dependency of the items was evaluated using a correlation analysis of the residuals of the items. A coefficient of above 0.2 or below − 0.2 was chosen to indicate that the responses were dependent on each other or not fitting the Rasch model [24].

Fit to the Rasch model was investigated for the items and the individual participants. The fit of the items was statistically evaluated using standardized residuals and chi-squared statistics according to the weighted maximal likelihood method with residuals <  ± 2.5 and a non-significant chi-squared probability accepted as a fit to the Rasch model. The overall summary fit of the RRTW working and not working scales as well as the subscales was evaluated using chi-squared item-trait interaction statistics (χ^2^). The probability level of 0.05 was Bonferroni-adjusted according to the number of items included in the analysis. A nonsignificant probability value indicates a fit to the Rasch model [25]. The Person Separation Index (PSI) was calculated for all scales and subscales to estimate the scale’s internal consistency. PSI is the Rasch-based analogue to Cronbach’s alpha [25]. The PSI also indicates of the power of the measure to discriminate among persons with different levels of a trait. A value above 0.8 was deemed to differentiate across at least three groups of subjects. Invariance across age (dichotomized into groups below and above the median age of 46 years) and gender was assessed using a differential item functioning (DIF) analysis. DIF was assessed by an analysis of variance for each item, comparing the scores across each level of gender and age. The targeting of the RRTW scale and subscales fitting the Rasch model was evaluated by examining the hierarchical distribution of the items/statements and their response levels and was compared to the distribution of the patients along the same metric scale. The Rasch analyses were performed in RUMM 2030 (RUMM Laboratory, Perth, Australia). We needed 100 persons to detect stable item and person estimates within ± 0.5 logit with a 95% CI [26]. Generally, 10 persons per response category are recommended and assuming a not perfectly even response distribution across the 5-point scaling, we estimated a need for at least 150 persons [27]. Hence, power was not high enough to run DIF by diagnostic group, but preliminary analyses did indicate invariance across diagnostic groups. Furthermore, exploratory t-tests were performed to explore differences between persons with musculoskeletal and mental health conditions for each subscale.

Linear and logistic regressions with adjusted *R*^2^ and pseudo *R*^2^ were used to assess how well Rasch-based scaling (logit person location values) predicted future sickness absence and sustainable RTW. The analyses were performed without adjustments and were adjusted for age, gender, education, rehabilitation program and length of sick leave at inclusion. Age was included as a continuous variable. Education was dichotomized as high (college/university) or low. As the linearity assumption was not satisfied, the analyses were repeated with the variable categorized (≤ 0.0, 0.01–1.00, 1.01–2.00, and ≥ 2.01). The regression analyses were performed using STATA 16 (StataCorp. 2019. Stata Statistical Software: Release 16. College Station, TX: StataCorp LP).

## Results

A total of 397 participants completed the RRTW scale (191 not working and 206 working (Table 1). Of the participants filling out the not working part of the questionnaire, 36% (*n* = 68) achieved sustainable RTW, and the median number of sickness absence days during 12 months of follow-up was 174 (25th–75th percentiles 103–229). Of those filling out the working part, 64% (*n* = 132) achieved sustainable RTW and the median number of days on sick leave was 68 (25th–75th percentile 36–123). Table 1 shows baseline characteristics for the two groups. No significant difference in RTW attitudes was revealed between the patients with musculoskeletal and common mental health problems in any dimension (*p* > 0.09).

**Table 1:** Baseline characteristics

	Not working^a^ (n = 191)	Working^b^ (n = 206)
Age mean (SD)	46 (8.5)	46 (8.7)
Women n (%)	150 (79)	171 (83)
Higher education n (%)^c^	81 (42)	107 (52)
Main diagnosis for sick leave (ICPC-2) n (%)		
A- General and unspecified	23 (12)	19 (9)
L- Musculoskeletal	88 (46)	106 (51)
P- Psychological	76 (40)	80 (39)
Missing	4 (2)	1 (0.5)
Length of sick leave at inclusion		
Mean (SD)	209 (75)	212 (71)
Median days (25th–75th percentiles)	206 (160–270)	206 (172–259)
Readiness for Return to Work		
Mean (SD)/median (25th–75th percentiles)		
Precontemplation (1–5)	1.6 (0.8)/1.0 (1.0–2.0)	
Contemplation (1–5)	3.9 (0.8)/4.0 (3.3–4.7)	
Prepared for action—self-evaluative (1–5)	2.5 (0.9)/2.5 (2.0–3.0)	
Prepared for action—behavioral (1–5)	3.6 (0.8)/3.7 (3.0–4.3)	
Uncertain maintenance (1–5)		3.5 (0.8)/3.6 (3.0–40)
Proactive maintenance (1–5)		3.8 (0.7)/4.0 (3.5–4.3)

^a^100% sick leave

^b^Part time sick leave and/or working

^c^College or university

### The Rasch Analysis

#### Not Working Sample

**Fig. 1:**
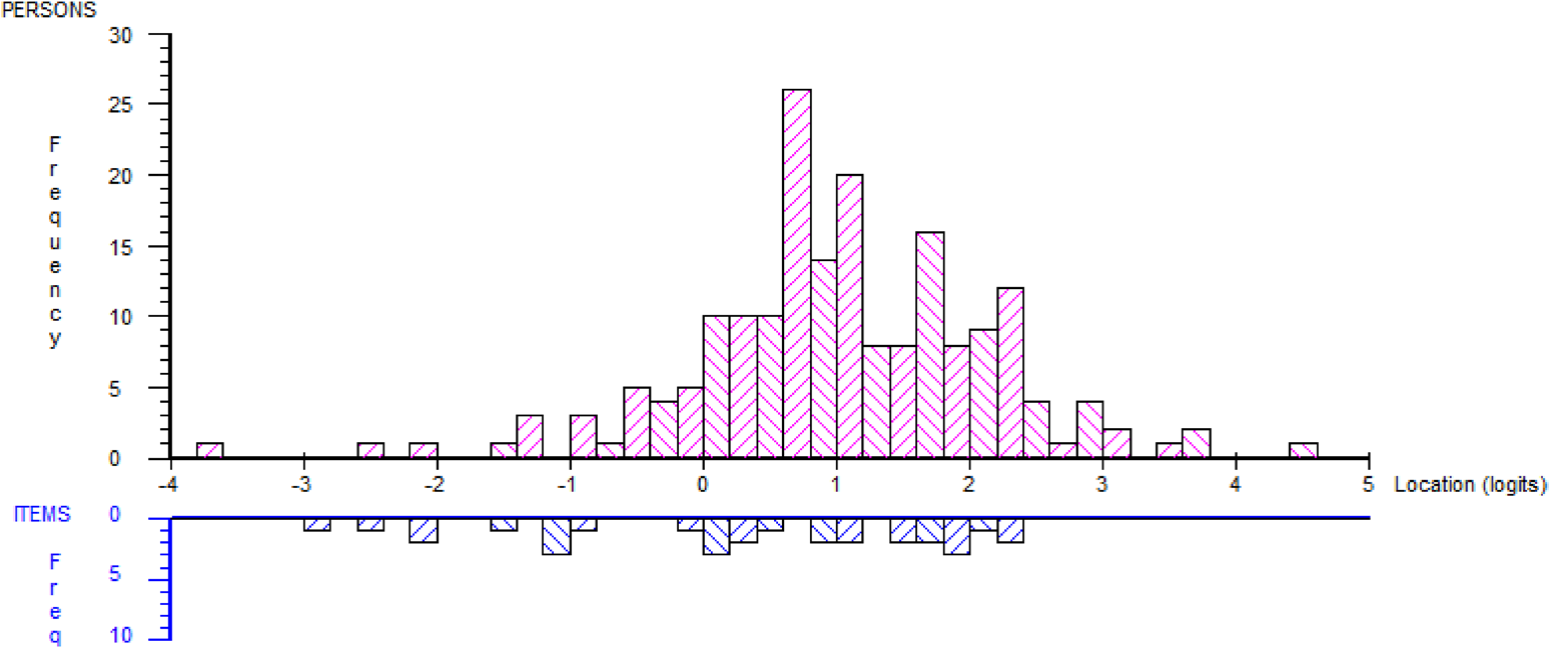
Distribution of scores and threshold for the 16 items of the Readiness for Return to Work scale fitting the Rasch model for the not working sample (*n* = 191). Items and persons are presented on the same logit scale. Mean location score for persons was 1.03 (SD 1.12), indicating that items in the present scale are targeted to persons with less positive attitudes towards return-to-work than the included persons

The Rasch analysis of the not working sample revealed disordered thresholds for all items except A9 and A14. These items were re-scored, retaining two to four scoring options for all items (Table 2). The 22 items of the not working sample of the RRTW scale did not fit the Rasch model, despite rescoring all the items with disordered thresholds (*χ*^2^ = 195.81, *p* < 0.001). Items A12, A15, A16, A19, A20, and A21 did not fit the model, but after removing these items, the remaining 16 items fit one unidimensional construct (*χ*^2^ = 42.32, *p* = 0.10). Residual correlations were observed between A1 and A4, A1 and A22, and A4 and A22 (> 0.6), whereas A7 and A8, A7 and A17, and A8 and A17 were correlated above 0.2. Hence, redundancy of some of these items was indicated. Fit of items was mean 0.17 (SD 1.01) and fit of persons was mean − 0.27 (SD 0.77). PSI was 0.80, indicating the ability to differentiate across three groups. Three subjects revealed extreme scores. Mean person location was 1.03 (SD 1.12), indicating that the subjects rated a higher likelihood of RTW than the items reflected. Few items reflected the subjects with a high likelihood for RTW, indicating a ceiling effect in this 16-item version (Fig. 1). The majority of the participants disagreed with the statement “As far as I’m concerned, I don’t need to go back to work ever”, whereas most of the participants reported to have a date for RTW (Online Supplementary Table 1).

**Table 2:** The 22 items in the not working subset (*n* = 191) of the Readiness for Return to Work (RRTW) scale

	Item score
A1 I don’t think I will ever be able to go back to work (PC)	1(1,2), 2(3,4,5)
A2 I have made a plan together with the workplace for return to work	1(1,2), 2(3,5)
A3 I have planned some changes that will help me return to work	1(1,2), 2(3,4,5), 3(4,5)
A4 As far as I’m concerned, there is no point in thinking about returning to work (PC)	1(1,2), 2(3,4,5)
A5 I have learned different strategies for coping with my health complaints in order to return to work	1(1,2), 2(3), 3(4,5)
A6 I am doing things actively now to get back to work (B)	1(1,2), 2(3), 3(4,5)
A7 I think I may be ready for return to work	1(1,2), 2(3,4), 3(5)
A8 I plan returning to work, even though I still have some health problems	1(1,2), 2(3,4), 3(5)
A9 Physically, I am starting to feel ready to go back to work (E)	1, 2, 3, 4, 5
A10 I have been increasing my activities at home in order to build up my strength to go back to work (B)	1(1,2), 2(3), 3(4,5)
A11 I am getting help from others to return to work (B)	1(1,2), 2(3), 3(4,5)
A12 I am not ready to go back to work (E)	1(1,2), 2(3), 3(4,5)
A13 I have found strategies to make my work manageable so I can return to work (E)	1(1,2), 2(3), 3(4), 4(5)
A14 Mentally I feel ready for return to work	1, 2, 3, 4, 5
A15 I have been wondering if there is something I could do to return to work (C)	1(1,2), 2(3), 3(4), 4(5)
A16 I worry about having to stop working due to my health complaints	1(1,2,3), 2(4,5)
A17 I have started to think about return to work	1(1,2), 2(3), 3(4), 4(5)
A18 I have a date for my first day back at work (E)	1(1,2), 2(3,4,5)
A19 I wonder if I will be able to return to work	1(1,2,3), 2(4,5)
A20 I wish I had more ideas about how to get back to work (C)	1(1,2), 2(3), 3(4,5)
A21 I would like to have some advice about how to get back to work (C)	1(1,2), 2(3,4), 3
A22 As far as I’m concerned, I don’t need to go back to work ever (PC)	1(1,2), 2(3,4,5)

The rescored values are presented while the original responses are placed in brackets. Items that are included in the subscales of Precontemplation (PC), Contemplation (C), Prepared for action—self-evaluative (E), and Prepared for action—behavioral (B) are indicated with abbreviations

Furthermore, separate analyses were performed for the dimensions of Precontemplation, Contemplation, Prepared for action—self-evaluative and Prepared for action—behavioral. Neither the Precontemplation nor the Contemplation subscale fit the Rasch model (*p* < 0.04), and negative residual correlations (< − 0.3) were observed among all items in both subscales. As these scales comprise only three items each, the power of the Rasch analyses was low. The Prepared for action—self-evaluative subscale fit the Rasch model (*χ*^2^ = 7.36, *p* = 0.50), but also in this subscale, negative residual correlations (< − 0.2) were observed among all items. The power of the analysis was also low, and the PSI of 0.47 indicates a lack of ability to differentiate across groups. Also, the Prepared for action—behavioral subscale fit the Rasch model (*χ*^2^ = 6.70, *p* = 0.08), but with low power, and the residual correlations among all items were negative (< − 0.2). Invariance across age and gender was revealed for all items in the not working sample.

**Fig. 2:**
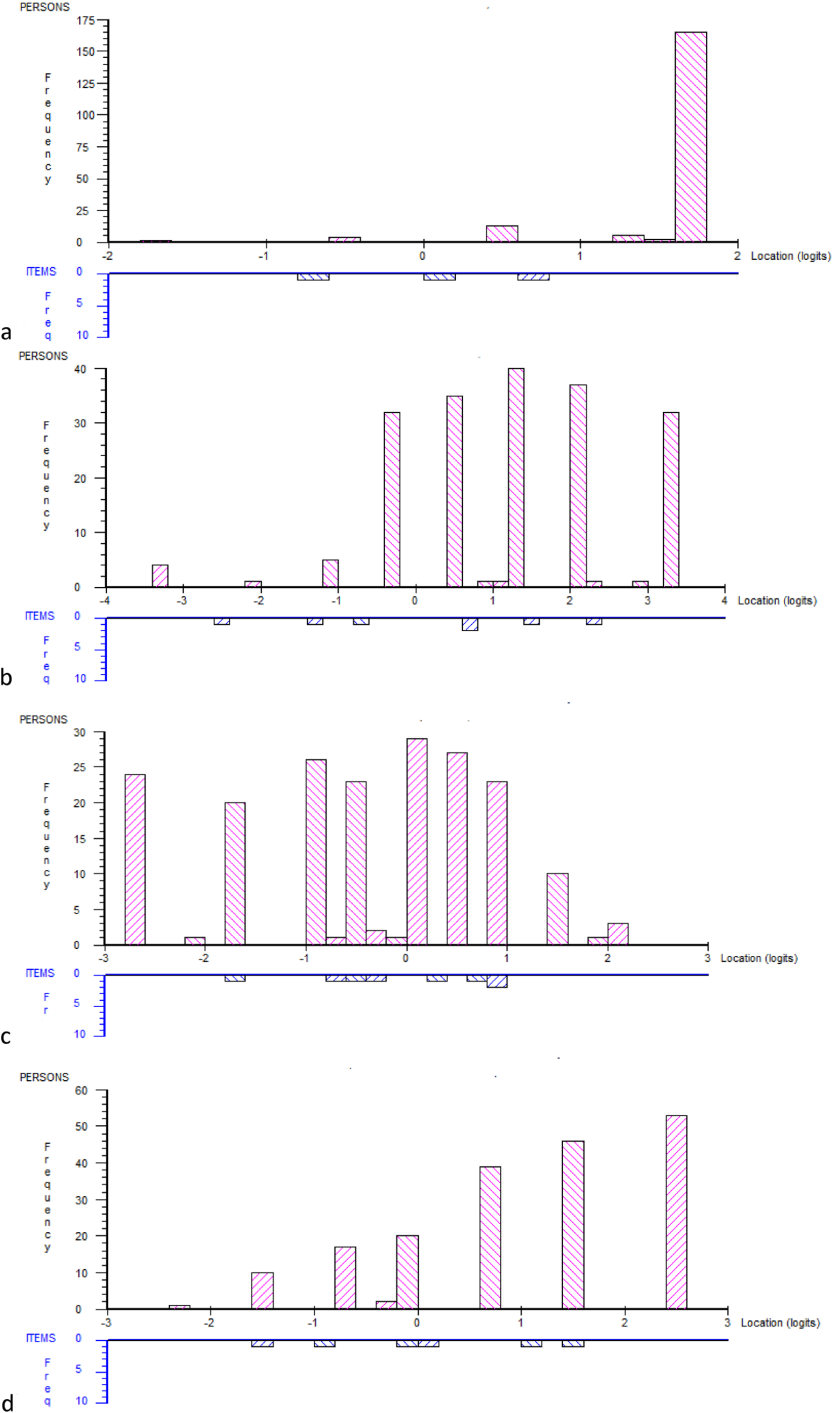
Distribution of scores and threshold for items and persons (not working sample *n* = 191) in the of the Readiness for Return to Work subscales: **a** Precontemplation, mean location score for persons 1.53 (SD 0.48), **b** Contemplation, mean location score for persons 1.22 (SD 1.39), **c** Prepared for Action—self-evaluative, mean location score for persons 0.10 (SD 0.90), and **d** Prepared for action—behavioral, mean location score for persons 1.03 (SD 1.21). The targeting was particularly poor for the Precontemplation dimension (**a**), where 174 subjects had extreme location score (maximal score), and thus much more positive to return-to-work than reflected by the items (ceiling effect). In the Contemplation dimension (**b**), 40 subjects revealed extreme location scores (4 low and 36 high), in the Prepared for action—self-evaluative dimension, 30 subjects revealed extreme location scores (26 low and 4 high), in the Prepared for action—behavioral dimension, 49 subjects revealed extreme location scores (3 low and 46 high)

Mean location scores for persons are provided in Fig. 2 for the Precontemplation, Contemplation, Prepared for action—self-evaluative, and Prepared for action—behavioral dimension. Given only two valid scoring options for the items in the Precontemplation dimension, this mean location score for persons, along with the number of extreme locations, indicates a substantial ceiling effect (or a floor effect, if the scale had not been reversed) in this dimension in the not working population (Fig. 2a). A ceiling effect was also indicated for the Contemplation dimension and the Prepared for action—behavioral dimension whereas for the Prepared action—self-evaluative dimension there was a floor effect. The correlations between the subscales were below 0.33 (Spearman’s rho, *p* < 0.03).

Furthermore, considering the ceiling effect in the Prepared for action—behavioral dimension, one would assume that the subjects with extreme locations also revealed high-level locations in the Prepared for action—self-evaluative dimension. However, only four of the 46 subjects with extremely high-level locations in the Prepared for action—behavioral dimension also revealed extremely high-level locations in the Prepared for action—self-evaluative dimension.

The new 16-item unidimensional construct was associated with future sickness absence days (adjusted mean − 8.8, 95% CI − 18.4 to 0.8) and sustainable RTW (OR 1.65, 95% CI 1.17 to2.33). However, the adjusted *R*^2^ and pseudo *R*^2^ were low (0.02 and 0.09, respectively). As the linearity assumption was not met, we repeated the analyses with the new construct as a categorical variable. There was then no association with sickness absence days. For sustainable RTW there seemed to be an association, but precision and explained variance were low (pseudo *R*^2^: crude 0.02, adjusted 0.07).

#### Working Sample

The Rasch analysis of the working sample of 12 items revealed disordered thresholds in all items except B3, B5, and B8. The nine items with disordered thresholds were rescored leaving 2 to 4 scoring options (Table 3). The 12 items of the working sample of the RRTW scale did not fit the Rasch model, despite rescoring all the items with disordered thresholds (*χ*^2^ = 92.43, *p* < 0.001), nor did the nine items included in the Uncertain and Proactive maintenance dimensions fit the Rasch model (*χ*^2^ = 59.76, *p* < 0.001). Items B8 and B11 revealed fit residuals above 2.5, but a lack of fit to the Rasch model remained after removing these items. Separating the Rasch analysis of the Proactive maintenance items (B2, B5, B6, and B7) and the Uncertain maintenance items (B8, B9, B10, B11, and B12) failed to support fitting to the Rasch models for any of the dimensions (*χ*^2^ = 21.20, *p* < 0.001; *χ*^2^ = 27.14, *p* < 0.001, respectively). DIF was revealed for age for item B9 with participants above 46 years who agreed with the statement “I worry about having to stop working again due to my health complaints” to a greater extent than younger participants relative to their general level of uncertain maintenance. Items B8 and B11 revealed DIF for gender with women who agreed more than had been expected with the statement “I am back at work but not sure I can keep up the effort” (B8) relative to their general level of uncertain maintenance. Men agreed more than had been expected with the statement “I am back at work and it is going well” (B11), relative to their general level of uncertain maintenance. None of the items correlated above 0.2 in the Uncertain maintenance and Proactive maintenance dimensions, whereas all items in both dimensions revealed negative item correlations (< − 0.2), indicating a lack of fit of the items to the Rasch model.

**Table 3:** The 12 items in the working subset (*n* = 206) of the Readiness for Return to Work (RRTW) scale

	Item score
B1 I try different strategies to continue working	1(1,2), 2(3,4), 3(5)
B2 I am doing everything I can to stay at work (P)	1(1,2,3), 2(4), 3(5)
B3 I have made a plan in collaboration with the work place for return to work	1,2,3,4,5
B4 From my point of view I should not consider to return to work	1(1,2),2(3),3(4),4(5)
B5 I have learnt different ways to cope with my health complaints so I can stay at work (P)	1, 2, 3, 4, 5
B6 I am taking steps to prevent having to go off work due to my health complaints (P)	1(1,2), 2, 3, 4
B7 I have found strategies to make my work manageable so I can stay at work (P)	1(1,2), 2, 3, 4
B8 I am back at work but not sure I can keep up the effort (U)	1, 2, 3, 4, 5
B9 I worry about having to stop working again due to my health complaints (U)	1(1,2,3), 2(4,5)
B10 I still find myself struggling to stay at work due to my health complaints (U)	1, 2,3,4(4,5)
B11 I am back at work and it is going well (U)	1, 2, 3, 4, 5
B12 I feel I may need help in order to stay at work (U)	1, 2, 3, 4(4,5)

The rescored values are presented while the original responses are placed in brackets. Items that are included in the dimensions of Uncertain maintenance (U) and Proactive maintenance (P) are indicated with abbreviations

Five subjects had extreme scores in the Uncertain maintenance dimension, and one with a missing score for this dimension (no items filled in). For the Proactive maintenance dimension 17 subjects had extreme scores. The correlation between the mean score for person location of the two dimensions was unexpectedly low (Spearman’s rho 0.21, *p* = 0.03). Mean location of the participants’ scoring on the five items in the Uncertain maintenance dimension was − 0.49 (SD 1.05) and 0.79 (SD 1.56) in the Proactive maintenance dimension. The negative mean location score for persons in the Uncertain maintenance dimension indicates that the subjects were more uncertain about maintaining work than the items reflected. In contrast to the expected results, the mean location score for persons in the Proactive maintenance dimension was positive, indicating a lower degree of items with proactive attitudes in this subscale compared to the persons. Analyses on the association with future work participation were not performed for the working group due to the lack of unidimensionality in the Rasch analyses; thus, we had no valid Rasch-based person-location scores to use for prediction. The item fit and location of the nine items included in the Uncertain and Proactive maintenance dimensions are presented in Online Supplementary Table 2.

## Discussion

Based on the Rasch analyses, the RRTW subscales had too few items to properly represent the underlying dimensions. The analyses also indicated that the items did not fit together within the subscales. Also, floor and ceiling effects were found, as well as unexpected responses across the subscales. A constructed variable based on the items that fit together for not working individuals poorly predicted future work participation.

The problems with the RRTW scale, identified via the Rasch analyses, corroborate previous studies that found problems with the questionnaire [5, 7]. None of the previous studies were able to identify all the original stages described by Franche et al. [3–6]. This is in line with the Rasch analysis results showing that the subscales consisted of too few items to constitute separate constructs. It has previously been suggested to look at the original pool of items—not only those included in the final questionnaire [3]. When we did this, it became possible to combine 16 items into a new construct for the not working group. However, this new construct was not a good predictor of future work participation.

The low number of items in the subscales is a challenge when trying to capture complex underlying constructs as RRTW. In the not working sample, the Precontemplation and Contemplation subscales did not fit the Rasch model. The Prepared for action—self-evaluative and Prepared for action—behavioral subscales fitted the Rasch model (p < 0.05), but the low power and extremely low PSI indicated that these subscales did not represent valid measurements. The targeting and extreme scores of these dimensions did not support a stage ordering of the dimensions and person scores: individuals that scored high on the Prepared for action—behavioral dimension did not score high on the Prepared for action—self-evaluative dimension. This could indicate that the items in the Prepared for action—behavioral dimension do not capture this dimension well, which would corroborate with our previous study where we found that high scores on the Prepared for action—self-evaluative dimension revealed stronger associations with future work outcomes than the Prepared for action—behavioral dimension [7].

Another problem in the not working sample was floor and ceiling effects. The ceiling effect in the Precontemplation dimension was not surprising; this study included participants who had agreed to occupational rehabilitation after long-term sick leave. A floor effect in this dimension has been reported previously, corresponding to the ceiling effect we found in the present study when applying reversed scaling [5]. Having just three questions and only two valid scoring options for these questions contributed to this effect. According to the stage allocation approach this may not be a problem; where persons reaching the ceiling in one stage may be moved to the next stage. However, from a measurement perspective, this subscale is inadequate. The ceiling effect in the Prepared for action—behavioral dimension was more problematic, and this is in line with previous studies finding poor distribution of individuals across stages, i.e., individuals are allocated to some of the stages to a larger degree than other stages [3, 7] and ties between (not necessarily adjacent) stages when using the stage allocation approach [4, 7]. The potential problem of tied scores between stages was described by Franche et al. [4] in the original validation study of the scale. They described the advantages of having one stage per person as outweighing the problem, as this enables designing and delivering stage-based interventions. However, this also assumes that the stages are ordered with an increasing probability of RTW, which the results of the present study do not support.

In the working sample, we were unable to fit the 12 items of the full version or the nine items of the original scale to the Rasch model, nor did the four Proactive maintenance items or the five Uncertain maintenance items fit the Rasch model. The negative residual correlations may have contributed to the lack of fit. Nevertheless, a uniform construct of Uncertain or Proactive maintenance attitudes for work could not be confirmed. Hence, sum scores for the nine items or the subscales will not be valid. This would seem to support using the stage allocation approach. However, the targeting and higher number of extreme scores in the Proactive maintenance dimension compared with the Uncertain maintenance dimension contradicts the stage allocation theory. These results are in line with our previous study showing that persons in the Uncertain maintenance stage had a higher probability of sustainable RTW and more work participation days than those in the Proactive maintenance stage, based on registry data [7].

A general problem with the RRTW scale is that not working and working individuals answer different items. To properly test the stage allocation approach, it needs to be determined whether sick-listed workers actually go through all six stages while their probability of RTW changes correspondingly. Although we had access to longitudinal data for the RRTW scale, we were unable to utilize it to follow individuals over time, as they change which part of the questionnaire they answer when transitioning from not working to working. Hence, a revision is necessary for the RRTW scale to be validated. We suggest that questions applicable to both working and not working individuals are selected and tested prospectively in mixed populations taking part in RTW programs.

Some differences should be noted between study participants and context in the present study and the original development and validation study of the RRTW scale. While Franche et al. [4] included participants with work-related back or upper extremity musculoskeletal disorders, we included participants with musculoskeletal and common mental health complaints. Previous studies have indicated considerable overlap in health complaints among these diagnostic groups [28–30], and there were no significant differences their RTW attitudes. Due to power challenges, DIF by diagnostic group was not included in the final Rasch analysis, but preliminary analyses indicated no major differences. Work type may also affect the RTW process, and the original study by Franche et al. [4] included about 40% blue-collar workers. Unfortunately, information about work type was not available for our study. However, our study included more women and more participants who had completed higher education than the Canadian study, which suggests the inclusion more pink- and white-collar workers. There was also a vast difference in length of sick leave at inclusion; participants in the Canadian study were included early after their injury (median = 14 days of sick leave), while our study’s participants had been sick listed considerably longer (median about 200 days), indicating a complex RTW process. Furthermore, there are major differences in the compensation systems between the two countries, which could affect the workers’ motivation or their need for a quick RTW process. The Canadian Workplace Safety and Insurance Board (WSIB) is an insurance-based system covering work-related injury and illness (up to 85% of the worker’s salary), while the Norwegian system is universal and covers all types of medically certified sick leave (100% for the first 12 months) for all workers. In a previous Norwegian study, Braathen et al. [3] suggested that the different cultural and patient settings could explain why they did not identify the original factor structure. Although several factors differ between the original study and the present study and could affect the pace of the RTW process, we propose that the measurement problems of the RRTW scale we have identified explain the poor predictive abilities of the scale, and why several studies have had problems identifying the original factor structure of the scale [3, 5–7].

### Strengths and limitations

This is the first time the RRTW scale has been evaluated according to modern measurement standards. A main strength of this study is its use of registry data for sick leave measurements, ensuring no missing data or recall bias. Another strength is the evaluation of the RRTW scale in a country other than Canada where it was developed. Some limitations should be addressed. First, some modifications were made to the wording of the original questionnaire. Changing the word *injury* was necessary because the word is not commonly used to describe unspecific musculoskeletal complaints like back pain in Norway. Furthermore, traditional Norwegian RTW interventions are not diagnosis specific, making it natural to replace the word *pain* with *health complaints*. We do not assume that these changes affected the scaling or difficulty of the items, yet we acknowledge the limitations related to the lack of formal validation. Second, the number of sickness absence days was not normally distributed for not working individuals. However, the analyses were also performed for sustainable RTW with similar results. It should also be noted that the interventions the participants received in the trials differed in terms of how they were designed to increase work ability. However, the Rasch analyses were based on data collected before the participants received the interventions.

## Conclusion

This study found several problems with the RRTW scale in individuals with musculoskeletal and common mental disorders. Both the scoring option intended for research and the option intended for clinical use, displayed poor measurement qualities. The RRTW subscales contained too few items to properly represent underlying dimensions, and the items that fit together as a construct poorly predicted future work participation. Furthermore, there were floor and ceiling effects and a disorganization of the subscales, which could explain previous problems regarding the poor predictive abilities of the scale and ties between the stages. Hence, in its current state, the Norwegian version of the questionnaire should not be used in the clinic or for evaluating interventions. Future research should evaluate whether the problems this study found with the RRTW scale also applies to other versions and for other populations.

## Supplementary Information

Below is the link to the electronic supplementary material.

Supplementary file1 (DOCX 20 kb)
