# Supplementary material for: The Readiness for Return to Work Scale; Does it Help in Evaluation of Return to Work?
Source: J Occup Rehabil. 2021 Oct 16;32(3):426–37. doi: 10.1007/s10926-021-10009-4 (PMC9576643; doi:10.1007/s10926-021-10009-4)
Supplement: Supplementary file 1 — Supplementary file1 (DOCX 20 kb) [file 10926_2021_10009_MOESM1_ESM.docx]

**Online supplementary table 1**

Location with standard error (SE) of the 16 items in the not working subset of the Readiness for Return to Work (RRTW) scale that fit in one unidimensional construct according to the Rasch analysis (*n*=191).

| Item | Location | SE | FitResid | ChiSq | Prob |
| --- | --- | --- | --- | --- | --- |
| A1 I don`t think I will ever be able to go back to work (PC) | -2.18 | 0.33 | 0.48 | 1.11 | 0.57 |
| A2 I have made a plan together with the workplace for return to work | 1.42 | 0.16 | 0.34 | 1.69 | 0.81 |
| A3 I have planned some changes that will help me return to work | -0.62 | 0.14 | 0.83 | 2.07 | 0.35 |
| A4 As far as I`m concerned, there is no point in thinking about returning to work (PC) | -2.53 | 0.37 | 1.33 | 4.34 | 0.11 |
| A5 I have learned different strategies for coping with my health complaints in order to return to work | 1.08 | 0.12 | 0.27 | 3.43 | 0.18 |
| A6 I am doing things actively now to get back to work (B) | -1.23 | 0.17 | -0.54 | 2.50 | 0.29 |
| A7 I think I may be ready for return to work | -0.33 | 0.15 | -1.02 | 1.98 | 0.37 |
| A8 I plan returning to work, even though I still have some health problems | 0.29 | 0.13 | -0.97 | 0.93 | 0.63 |
| A9 Physically, I am starting to feel ready to go back to work (E) | 1.36 | 0.08 | 0.38 | 1.60 | 0.45 |
| A10 I have been increasing my activities at home in order to build up my strength to go back to work (B) | 0.56 | 0.11 | -0.32 | 1.70 | 0.43 |
| A11 I am getting help from others to return to work (B) | 0.36 | 0.11 | 2.67 | 5.64 | 0.06 |
| A13 I have found strategies to make my work manageable so I can return to work (E) | 0.89 | 0.16 | -0.49 | 4.41 | 0.11 |
| A14 Mentally I feel ready for return to work | 1.23 | 0.08 | -0.37 | 0.38 | 0.83 |
| A17 I have started to think about return to work | 0.45 | 0.14 | -1.11 | 3.54 | 0.17 |
| A18 I have a date for my first day back at work (E) | 2.21 | 0.18 | -0.11 | 2.78 | 0.25 |
| A22 As far as I`m concerned, I don`t need to go back to work ever (PC) | -2.97 | 0.44 | 1.29 | 5.48 | 0.06 |

PC= Precontemplation; C= Contemplation; E= Prepared for action - self-evaluative; B= Prepared for action – Behavioral; ChiSq= Chi square; FitResid= Fit residual; Prob= probability.

**Online supplementary table 2**

Location with standard error (SE) of the 9 items in the original subscales of the working subset of the Readiness for Return to Work (RRTW) scale (*n*=206).

| Item | Location | SE | FitResid | ChiSq | Prob |
| --- | --- | --- | --- | --- | --- |
| B2 I am doing everything I can to stay at work (P) | -0.94 | 0.11 | 0.22 | 4.60 | 0.10 |
| B5 I have learnt different ways to cope with my health complaints so that I can stay at work (P) | -0.45 | 0.086 | -0.07 | 0.51 | 0.77 |
| B6 I am taking steps to prevent having to go off work due to my health complaints (P) | -0.98 | 0.10 | 0.20 | 1.51 | 0.47 |
| B7 I have found strategies to make my work manageable so I can stay at work (P) | 0.11 | 0.09 | 0.40 | 2.38 | 0.30 |
| B8 I am back at work but not sure I can keep up the effort (U) | 0.29 | 0.08 | 3.31 | 16.86 | <0.01 |
| B9 I worry about having to stop working again due to my health complaints (U) | 1.07 | 0.17 | -0.57 | 5.68 | 0.60 |
| B10 I still find myself struggling to stay at work due to my health complaints (U) | -0.12 | 0.09 | -0.75 | 2.95 | 0.23 |
| B11 I am back at work and it is going well (U) | 0.55 | 0.08 | -2.02 | 19.78 | <0.01 |
| B12 I feel I may need help in order to stay at work (U) | 0.47 | 0.09 | 1.32 | 5.49 | 0.06 |
|  |  |  |  |  |  |

P= Proactive maintenance contemplation; U= Uncertain maintenance; FitResid= Fit residual; ChiSq = Chi square; Prob= probability
